# Learning the language of plant immunity: opportunities and challenges for AI-assisted modelling of fungal effector x host protein complexes

**DOI:** 10.1016/j.csbj.2025.06.048

**Published:** 2025-07-01

**Authors:** C. Verdonk, KK Gagalova, S. Raffaele, MC Derbyshire

**Affiliations:** a Centre for Crop and Disease Management, Curtin University, Perth, Australia; b Université de Toulouse, INRAE, CNRS, Laboratoire des Interactions Plantes Micro-organismes Environnement (LIPME), Castanet-Tolosan, France

**Keywords:** Effector, Protein structure, Protein interaction, Plant receptor, Plant pathogen, AI structure prediction

## Abstract

Phytopathogenic fungi cause substantial crop losses worldwide. They secrete proteins called effectors, which enable infection through interactions with diverse host proteins. These interactions are fundamentally important to plant disease and its practical control. New artificial intelligence (AI) techniques can predict many individual protein structures to near experimental accuracy. Although these techniques also predict protein complexes, they are not as accurate as single-protein models. Use of AI to study interactions between fungal pathogen effectors and plant proteins is currently limited. However, despite some challenges, early adoption of AI has highlighted its potential. General improvements in AI-assisted protein complex modelling may create more opportunities in future. This review focuses on recent research using AI to study the interactions between fungal effectors and plant proteins, outlining challenges and emerging opportunities.

## 1 Introduction

Proteins are among the most complex molecules in the known universe, and there is a fundamental need to understand their structure-function dynamic. Application of AI to prediction of protein structures from amino acid sequences has transformed the life sciences. Beginning with AlphaFold2 [1], several AI models can now predict many protein structures with near experimental accuracy [2], [3], [4], [5]. These disruptive techniques may help address many of the world’s greatest challenges.

However, the accuracy of predicted structures varies, with some high confidence structures containing local and global distortions [6]. Though the sudden abundance of protein structures has accelerated functional inference and classification, their interpretation without clear biochemical context can be misleading [7], [8]. A key question is how can the mass of predicted structures be funneled into targeted, biologically meaningful research?

Protein-protein interactions (PPIs) are among the most important biochemical events within cells and are required for myriad functions from homeostasis to stimulus response and disease progression [9]. Experimental techniques for understanding PPIs are often laborious, expensive and technically challenging. Although some experimental techniques can screen thousands of proteins, they are limited to testing interactions with a few probes, and cannot directly identify interaction interfaces [10]. AI-based structural modelling could therefore enhance PPI research by substantially increasing the throughput of novel PPI and interface detection.

Crop diseases caused by fungi significantly impact agriculture, reducing global crop production by an estimated 10–23 % per year [11], [12], [13]. Fungal plant pathogens rely on specialised secreted proteins called ‘effectors’ to cause disease [14]. PPIs between effectors and diverse plant proteins are central to infection and understanding them can provide new ways of managing crop diseases [15], [16]. For simplicity, we use the term ‘effector x host protein complex’ (EHPC) to refer to any complex containing a fungal pathogen effector and host plant protein. For a general review of the use of AI in modelling fungal effectors, the reader is directed to Jones and Raffaele (2025) [17]. For a broad recent review of *de novo* PPI prediction using machine learning, the reader is directed to Gainza et al. (2025) [18].

In this review, we provide background on EHPCs formed during fungal infections of plants and major AI techniques for modelling protein complexes. We then summarise the few studies representing a nascent trend towards AI-assisted EHPC research, highlighting limitations and opportunities. We conclude by discussing emerging themes in AI that may create new opportunities to deepen our understanding of PPIs at the molecular interface between plant and pathogen.

## 2 The diverse interactions between pathogen effectors and host proteins involved in immunity

### 2.1 Plant immune receptors detect conserved microbial molecules

Plants encounter numerous and diverse microbes throughout their lives. The plant immune system has evolved to monitor these microbes and stop pathogens from causing infections. It operates extracellularly via pattern-recognition receptors (PRRs), typically either receptor-like proteins (RLPs) or receptor-like kinases (RLKs). PRRs typically recognise microbe-associated molecular patterns (MAMPs) and damage-associated molecular patterns (DAMPs). The former are molecular motifs broadly conserved in microbes and the latter are host-derived molecules produced under pathogenic conditions [19], [20], and both indicate threat of invasion by a foreign organism [21].

RLPs and RLKs initiate a basal immune response known as pattern-triggered immunity (PTI), which is characterised by a swift burst of reactive oxygen species (ROS), calcium (Ca^2 +^) influx and initiation of plant hormone-mediated signalling cascades [22]. The rapidly produced ROS both mediate immune signalling and form an antimicrobial barrier to pathogen proliferation [23]. For a recent review of PTI, readers are directed to Shu et al. (2023) [24].

PTI signalling cascades activate downstream defences that limit pathogen invasion of host cells. These may include deployment of physical barriers through cell wall remodelling, callose deposition, lignification and stomata closure [25], or antimicrobials, such as phytoalexins, pathogenesis-related (PR) proteins, antimicrobial peptides, such as defensins and thionins, [26], [27] and hydrolytic enzymes that degrade pathogen molecules [28], [29]. Some hydrolytic enzyme products are also DAMPs, which elicit further immune responses [28].

### 2.2 Plant immune receptors detect pathogen effector activities

Plants have evolved a second, stronger defence response called ‘effector-triggered immunity’ (ETI). This is typically activated by intracellular receptors in the nucleotide-binding leucine-rich repeat (NLR) family. ETI often causes a hypersensitive response (HR), involving localised programmed cell death (PCD). For a recent review of ETI, readers are directed to Remick et al. (2023) [30].

The PCD caused by ETI limits the spread of biotrophic pathogens, which require living host tissue. The classical zig zag model of plant immunity posits that biotrophic pathogens have evolved effector proteins that circumvent PTI. Plants have evolved intracellular receptors, usually in the NLR family, that recognise effectors to induce ETI. When the pathogen expresses an effector and its host a receptor that recognises it, ETI ensues, and the interaction is described as incompatible. Pathogen effectors that elicit ETI are described as avirulence (Avr) proteins and their cognate plant receptors as resistance (R) proteins [31]. When discussing incompatible interactions, we use the term ‘Avr protein’ for the effector and simply ‘effector’ otherwise.

The contingency of incompatibility on the presence of a cognate Avr-R protein pair gave rise to the ‘gene for gene’ model of plant pathogen interactions. However, PCD is ineffective against necrotrophic pathogens, which can thrive in dead host cells. These pathogens have evolved effectors that elicit PCD, often by interacting with NLR proteins to induce an ETI-mediated HR. For these pathogens, aggressiveness may be strongly influenced by the presence of a cognate pathogen effector host susceptibility (S) gene pair, giving rise to the ‘inverse gene for gene’ model of plant pathogen interactions [32].

Although ETI and PTI were initially described using the zig zag model as discrete layers of the plant immune system, they are now known to share signalling pathways and potentiate and amplify one another. The boundary between the two response systems is also blurred by the observation that PRRs can also elicit ETI [20], [22], [33]. New models, such as the ‘invasion’ [34] and ‘spatial’ models [20], have been proposed to better-describe the plant immune system. These emphasise the location of the immune response within the plant cell.

### 2.3 Beyond effector-immune receptor pairs

As gene-for-gene interactions began to be studied, many failed to demonstrate direct interactions between Avr proteins and their cognate R proteins. Furthermore, many R proteins were found to recognise multiple structurally unrelated Avr proteins. These observations gave rise to the guard model, which proposes that R proteins often recognise Avr proteins indirectly [35], [36]. The core principle of this model is that the Avr protein, an effector, interacts with a protein in the host to facilitate infection. By guarding the targets of effectors, R proteins monitor modifications induced by effector binding.

However, this presents an evolutionary conundrum where the effector target, or ‘guardee’, is subjected to opposing selective pressures. In the absence of the R protein, alleles preventing the effector from binding the guardee are advantageous as they reduce susceptibility. However, when the R gene is present, they prevent recognition, becoming disadvantageous. In many interactions, the guardee is thought to be a non-functional analogue of the original Avr protein’s target, giving rise to the ‘decoy’ model of R protein function [37], which provides an answer to this evolutionary paradox.

In addition, many NLR proteins are now known to contain non-canonical domains homologous to the targets of the Avr proteins they respond to. These integrated decoy domains (IDDs) act as effector target mimics, and are present among 10–15 % of the NLRs encoded in many plant genomes [38]. A common mechanism for NLRs may be formation of a heterodimer containing an NLR with an IDD that directly binds an Avr protein and an NLR without an IDD that triggers an immune response [39], [40].

### 2.4 How the plant immune system realys the signal from recognition to defence

Both ETI and PTI signals are relayed from receptors to downstream defence genes by mitogen-activated protein kinase (MAPK) proteins [41]. RLKs are transmembrane proteins with varied extracellular ectodomains that recognise different molecular patterns and conserved intracellular kinase domains that typically phosphorylate proteins upstream of a MAPK cascade. Although RLPs also have extracellular ectodomains, they lack intracellular kinase domains and must associate with kinase-competent RLKs to initiate PTI signalling [42]. NLRs also rely on MAPK cascades, which are generally sustained for much longer during ETI than during PTI [43].

Plant immune responses are associated with substantial transcriptional reprogramming, often via key defence-associated transcription factors, such as those in the WRKY, MYB. AP2/ERF, bZIP, NAC and bHLH families [44], [45]. Activation of plant immunity is also associated with broad epigenetic reprogramming. For instance, increased expression is correlated with increased chromatin accessibility among more than one third of genes induced during PTI and a combination of PTI and ETI [46], [47]. Furthermore, chromatin remodelling complexes have been shown to play a key role in plant defences by modifying nucleosome positions with respect to defence genes [48].

**Fig. 1:**
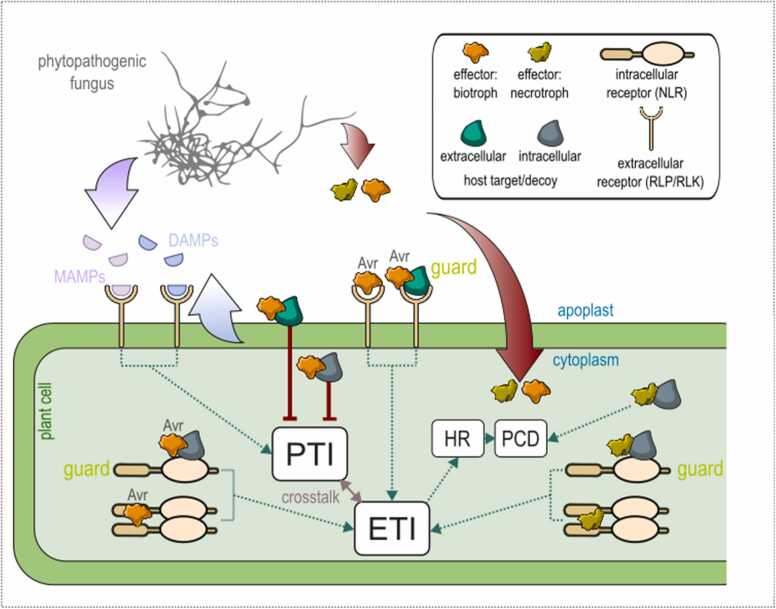
Fungal effectors and plant immunity**.** Conserved microbe-associated molecular patterns (MAMPs) and damage associated molecular patterns (DAMPs) trigger pattern-triggered immunity (PTI) via cell surface RLP and RLK receptors, leading to downstream defences. Many fungal effectors suppress PTI or inhibit downstream defences by binding to extra and intracellular host targets. Biotrophic effectors are detected by nucleotide-binding leucine-rich repeat (NLR) proteins, activating effector-triggered immunity (ETI), which triggers a ‘hypersensitive response’ (HR) leading to programmed cell death (PCD). ETI may also be initiated by recognition of biotrophic effectors by cell surface receptors in the apoplast. Necrotrophs, which feed on dead tissue, secrete effectors that trigger PCD to the pathogen’s benefit. This can occur via interaction with various host targets, including NLRs. Both NLRs and receptors at the cell surface, such as RLPs and RLKs, can recognise effector activities directly and indirectly through the proteins that effectors bind, which can be functional proteins or decoys.

Thus, the plant immune response is mediated by a complex integrated network whose output may be influenced by enumerable potential PPIs between host proteins and pathogen effectors. Fungal pathogens have evolved effector proteins to manipulate the plant immune system at various points from activation through to downstream defence outputs [49], [50] (Fig. 1).

### 2.5 The complex relationships between fungal effectors and the plant immune system encapsulated by interactions with plant secreted hydrolases

Most fungal effectors are small secreted proteins that target host proteins directly or indirectly in the cytoplasm or apoplast, including the xylem [51], [52], [53], [54]. Apoplastic effectors are typically cysteine-rich, whereas cytoplasmic effectors often contain more positively charged amino acids [55]. Coevolution with host proteins often makes effectors highly sequence diverse, although they are also often members of a few major structural groups [56], [57].

One of the effector mechanisms most extensively studied using AI is inhibition of plant secreted proteases. Tomato (*Solanum lycopersicum*) secretes numerous proteases that both degrade pathogen proteins and trigger plant immune signalling [58]. To counter these, the pathogen *Cladosporium fulvum* secretes the effector Avr2, which inhibits the tomato cysteine protease Pip1 by binding to its active site [59], [60], [61]. Clearly illustrating the decoy model of plant pathogen interactions, Avr2 also binds to the hydrolase Rcr3, which is monitored by the protein Cf-2. Upon formation of a complex between Avr2 and Rcr3, Cf-2 initiates an HR resulting in localised PCD. However, Cf-2 is a cell-surface localised RLP rather than an NLR, which underscores the blurred boundary between ETI and PTI response pathways [62], [63].

## 3 Modelling protein complexes with AI

AI-assisted structural prediction uses neural networks that relate amino acid sequences to protein structures. Whereas methods like AlphaFold and RoseTTA fold [5], [64] rely on evolutionary information from multiple sequence alignments (MSAs) or homologous templates, protein language models (pLMs) like ESMFold are trained to predict missing amino acids within sequences. These models uncover subtle, hidden relationships among amino acids that encode structural and evolutionary information, enabling accurate structure prediction without MSAs [5].

AlphaFold2 initiated a step-change in protein structure prediction accuracy [1]. Soon after, it was tailored specifically to PPIs to create AlphaFold-multimer. The AlphaFold2 model was since re-worked to create AlphaFold3, which improves on AlphaFold-multimer, predicting broadly reliable structures for around 70–80 % of tested protein complexes [65].

Recent research has simultaneously focused on *de novo* design of new protein structures. Building on RoseTTA fold, RFdiffusion is trained to iteratively denoise scrambled protein structures towards their native states. This training objective allows RFdiffusion to generatively produce structures likely to bind a given protein [66]. Similar capabilities are described for ESM3, which is trained to predict missing amino acids, structural coordinates and free-form functional descriptions of proteins [67].

Though AI models may approximate the biophysical energy function governing protein folding [68], [69], some studies suggest their understanding of it is limited [70]. Predicted structure inaccuracies may relate to this imperfect understanding and are relevant to both monomers and complexes. Furthermore, although PPI modelling is improving rapidly, AI techniques are not as accurate for protein complexes as for monomers [71]. General advances in AI-assisted complex modelling may therefore enhance research into EHPCs.

## 4 Early discoveries from AI-assisted research into effector host protein interactions

To date, a limited number of studies have used AI to investigate EHPCs. These studies have mainly focused on two broad effector activities, inhibition of secreted plant hydrolases and interaction with NLRs. AI contributions range from minimal support of experimental evidence [72] to high throughput PPI screens [73]. These studies highlight the limitations and potential of AI-assisted PPI analysis for enhancing our understanding of the molecular interactions between plants and fungal pathogens.

We highlight recent studies that demonstrate how structural prediction and AI-guided modelling have been applied to effector-receptor interactions, providing a concise overview of their applications and methodologies (Table 1).

**Table 1:** Recent studies using AI to model interactions between fungal pathogen effectors and plant host proteins.

**Research focus**	**Approach**	**Reference**
*Magnaporthe oryzae* x *Oryza sativa* (rice) NLR-effector pairs.	Used AlphaFold-multimer and 3 to model interactions between plant resistance proteins and pathogen effectors to identify direct Avr-R protein interactions.	Wang et al. (2024) [74]
All 93 known interacting pairs of NLR and effector proteins.	Assessed the properties of AlphaFold-multimer models of known and ‘forced’ NLR-effector pairs and used machine learning to distinguish between the two.	Fick et al. (2024) [75]
RD21-like papain-like cysteine-proteases from six plant species and protease inhibitor effectors from 13 pathogen species.	Assessed the confidence of AlphaFold-multimer models of known pairs of interacting effectors and host proteins, derived from Homma et al. (2023) [73].	Huang et al. (2024) [76]
*Puccinia striiformis* f. sp. *tritici* x *Triticum aestivum* (wheat) effector-receptor interactions.	Identified and characterized a DPBB domain-containing effector that interacts with host defence proteins using AlphaFold3 and molecular experimental techniques.	Asghar et al. (2025) [72]
Predicted small secreted proteins (SSPs) from seven *Solanum lycopersicum* (tomato) pathogens and tomato defence-associated secreted hydrolases.	Screened for PPIs among 11,274 pairs of proteins selected from combinations of 1879 pathogen SSPs and six defence-associated tomato hydrolases. Followed-up on highest confidence targets with experimentation.	Homma et al. (2023) [73]

### 4.1 Inhibition of plant secreted hydrolases

Plant secreted hydrolases degrade pathogen molecules and trigger immunity. Many fungal effectors inhibit these proteins by binding to their active sites. For instance, the biotrophic wheat pathogen *Puccinia striiformis* secretes an effector with a double-psi beta barrel (DPBB) fold that likely acts as a papain-like inhibitor of a secreted wheat cysteine protease. This has recently been implicated in immune suppression through its activity in multiple plant cell compartments, supported by co-immunoprecipitation assays and AlphaFold3 EHPC models [72].

Interaction with cysteine proteases was initially detected through immunoprecipitation of the DPBB effector from *Nicotiana benthamiana* leaves followed by mass spectrometry. However, AlphaFold3 models of complexes of the effector and each of the co-purified candidates were of low confidence. A higher confidence model was produced of a complex with a wheat orthologue of one target [72].

Low confidence models could arise from poor sequence conservation or intrinsic disorder rather than a lack of interaction [65]. The lack of congruence between an experimental technique and AlphaFold3 models highlights this limitation of AI-assisted EHPC analysis [72]. Furthermore, effectors and host proteins may only interact in complexes containing other proteins or multiple sub-units of the effector, receptor or both. This is impossible to determine without *a priori* knowledge of the complex. Therefore, it is important not to discard new candidate EHPCs identified with experimental techniques based solely on AI model quality.

**Fig. 2:**
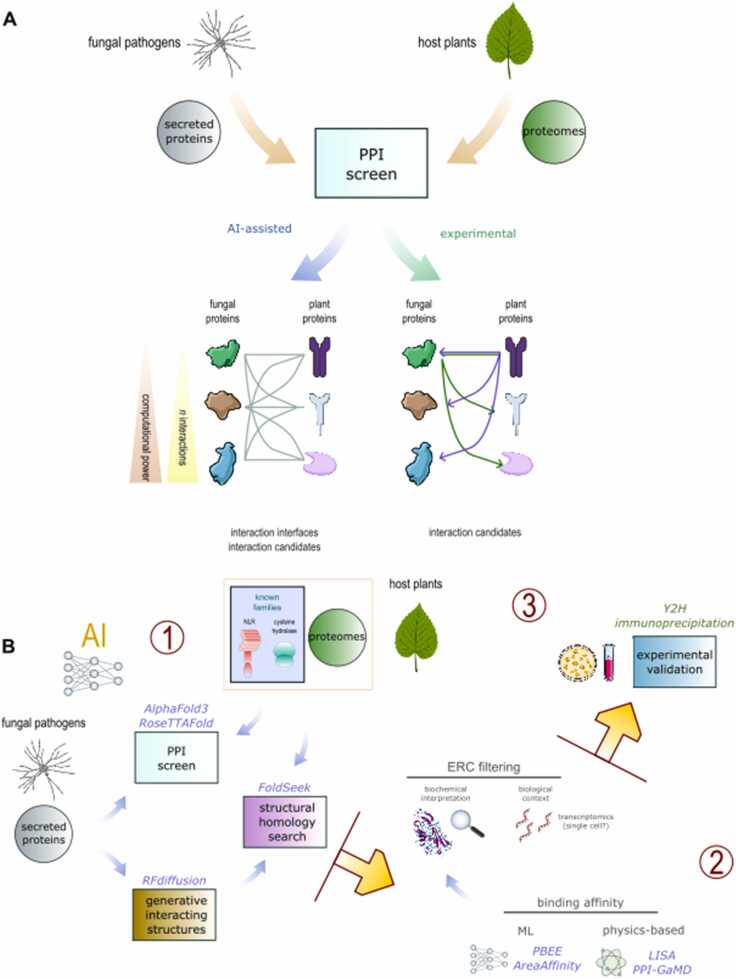
The difference in throughput between AI and experimental protein-protein interaction (PPI) screens.**A** Experimental techniques are limited to screening one or a few proteins against many targets, e.g. one candidate effector against a host proteome (bottom right, green arrows) or one host receptor against a fungal secretome (bottom right, purple arrows). AI modelling could screen for interactions between all possible pairs of candidate effectors and host proteins from multiple pathogen and plant species, simultaneously predicting interaction interfaces (bottom left). The number of comparisons is limited by available compute resources. **B** The broad approach outlined in this review includes: (1) detection of PPIs between whole host plant proteomes or a selection of known host targets involved in immunity and predicted secreted proteins from fungal pathogens using AI tools such as AlphaFold3 and RoseTTAFold. Alternatively, predicted secreted proteins from fungal pathogens may be used to generatively predict structures that they are likely to bind to; these generatively predicted structures may be searched against structures in plant proteomes using tools such as FoldSeek to detect potential interacting host proteins. (2) Expert biochemical and biological interpretation of candidate PPIs is essential for refinement of candidates for experimentation. Further information may be derived from transcriptomics or physics-based modelling techniques. (3) Experimental validation of PPIs using techniques such as yeast 2 hybrid or immunoprecipitation.

Experimental PPI identification followed by AI-assisted interface analysis is also limited by experimental throughput. Instead, initiating research with an AI-assisted PPI screen could broaden investigations to a vast number of potential PPIs and simultaneously predict binding interfaces, with an upper limit defined by available compute resources (Fig. 2). However, based solely on model quality, the biological and biochemical likelihood and significance of each complex in a large heterogeneous pool of potential PPIs may be difficult to decipher. This may challenge interpretation and experimental refinement.

This approach was used to screen 1879 small secreted proteins (SSPs) from seven tomato pathogens, including three fungal species. AlphaFold-multimer detection of potential PPIs between these SSPs and six defence-related secreted hydrolases from tomato led to the discovery and experimental validation of two new secreted plant protease inhibitors, Ecp36 and Six15 from *Cladosporium fulvum* and *Fusarium oxysporum*, respectively [73].

Crucially, candidate EHPCs were selected for experimentation based on their biochemical and biological features. For instance, non-sensical models where host and pathogen polypeptide strands were intertwined were excluded, as such complexes are unlikely for separately folded proteins secreted by different organisms [73]. Furthermore, to find novel EHPCs, models containing homologues of known protease inhibitors were also removed, and transcriptomic data were used to support effector expression during infection.

Important considerations for large-scale AI-assisted PPI screens are outlined in a follow-up study [77]. Again, limitations arising from MSA dependence are emphasized, as sparse homology for Avr2 leads to low confidence models for the known Avr2 Pip1 complex (described above). Furthermore, missense mutations in the oomycete effector EpiC2B known to abolish its interaction with Pip1 did not reduce the confidence of AlphaFold models of the EpiC2B-Pip1 complex [77].

As well as increasing throughput, AI modelling could also help identify binding interfaces for effectors and receptors with known interactions. This would bypass costly and time-consuming experiments helping to efficiently guide research. To this end, a recent study used experimentally determined structures from the protein databank (PDB) and AlphaFold-multimer to identify diverse binding interfaces between plant RD21-like papain-like cysteine proteases and interacting effectors from 13 pathogen species. High quality models were produced for four effectors, including *Mo*Ers1 from the rice blast fungus *Magnoporthe oryzae* [76].

### 4.2 Activation of effector-triggered immunity

Plant R proteins provide complete resistance against pathogen strains expressing corresponding Avr proteins, making them significant to disease resistance breeding. Extensive research has created catalogues of known interacting Avr and R gene pairs for many plant-pathogenic fungi. Identifying binding interfaces for proteins encoded by these pairs and high-throughput detection of new interactions is a major opportunity for AI-assisted EHPC modelling.

More than 40 *M. oryzae* Avr proteins have been identified, including several associated with cognate rice (*Oryza sativa*) R proteins. Using AlphaFold3 and AlphaPulldown [78] (a wrapper for AlphaFold2), potential interaction interfaces between three known Avr and five known R proteins have been found [74]. Echoing what was shown for Avr2 and Pip1, the known interaction between *M. oryzae* AVR-Pi9-and *O. sativa* Pi9 was confidently modelled by AlphaFold3 but not AlphaFold-multimer. This further emphasises the improvements in EHPC modelling that may derive from general AI performance gains for PPI analysis.

The broader properties of AlphaFold-multimer Avr R protein complex models have also been investigated. Across three known complexes, 80 % of NLR residues interacting with Avr proteins were shared between high confidence models and experimentally determined structures. However, Avr protein residues interacting with the NLR were generally less accurately predicted [75].

The biochemical features of a set of 58 known interacting Avr-R protein pairs and 2427 “forced” pairs from this set with no demonstrated interaction have also been investigated [75]. Whilst genuine and forced pairs had similar average binding affinities, the range of affinities was greater for forced pairs, one of which had the strongest overall affinity. This could reflect significant binding potential among forced pairs, despite them being unobserved, and in many cases unlikely, to interact in nature.

Biological relevance, then, is an important consideration during AI-assisted detection of new EHPCs. Although an effector and host protein pair may produce a confident complex model, it is only biologically relevant if both proteins are present at the same time and place, and relevant co-factors are present for complex formation and activity. This is especially important for NLR proteins, which often assemble into homomeric multimers and form complexes with other proteins, including NLRs [79], [80], [81], [82], [83], [84], [85]. NLR expression is also tissue-specific, with expression biased towards different tissues in different plant families [86]. Furthermore, given the indirect interactions between NLRs and effectors through decoy proteins or other NLRs carrying IDDs, restricting AI modelling to direct interactions between NLRs and effectors may limit potential for discovery.

The biological and biochemical context of PPIs is also a challenge to AI-based PPI modelling. The impact of transient interactions, local host-protein cellular environment and co-factor dependencies are often overlooked by isolated AI-generated models which generally capture static interactions in their modelling algorithms. Additionally, many AI models are trained on incomplete or biased datasets, which disproportionately represent well-studied organisms or protein complexes, possibly limiting generalizability across diverse biological systems.

Computational estimation of binding affinity could assist prioritization of candidate EHPCs for experimentation. Simple physics-based tools such as LISA [87] have been shown to accurately estimate binding affinity for PPIs. Computationally demanding molecular dynamics simulation tools, such as PPI-GaMD may provide a more detailed understanding of the biochemical properties of PPIs, including their binding affinities [88]. Machine learning (ML) methods, such as PBEE [89] and AreaAffinity [90], have been shown to predict binding affinity with an accuracy rivalling that of the most accurate physics-based methods. ML techniques use fewer computational resources than the most accurate physics-based methods, so may be useful for predicting binding affinity for a larger number of candidate EHPCs (Fig. 2).

Other studies have used AI in different ways to improve detection of genuine NLR-effector interactions. Large language models trained on vast datasets embed sequence inputs into a latent vector space that carries extensive evolutionary and structural information. Leveraging these embeddings as input for bespoke classification models is a promising approach for developing accurate predictions in low data settings. Application of this technique to 387 corresponding NLR effector protein pairs showed promise for distinguishing between genuine and forced interactions [91]. However, the complexity of NLR-effector interactions, which are often indirect, and development of a meaningful negative training dataset remain challenging.

Overall, although AI-assisted PPI analysis has potential for high throughput detection of new Avr-R protein pairs and interaction interfaces, as for other EHPCs, biological and biochemical interpretation, and complementary experimental validation, remain crucial for determining their relevance and accuracy. Careful consideration must also be given to biological context, including understanding the abundance of the investigated effectors and/or the receptor proteins within the host, and further physiological impacts such a PPI has on fungal pathogenicity and plant host response.

## 5 Looking to the future: trends in AI protein structure modelling that will impact research on effectors and their receptors

AI PPI modelling techniques are rapidly evolving and may soon create new opportunities for analysing EHPCs. Several studies using AI to model EHPCs have relied on AlphaFold-multimer [92], a model similar to AlphaFold2 adapted to PPIs. However, AlphaFold3 [65] represents a significant advancement in structural biology, offering improved accuracy in predicting not only protein structures but also their interactions with other biomolecules, including antibodies. This enhancement is particularly relevant for modelling rapidly evolving pairs of effectors and receptors, which often pose challenges due to limited sequence homology [56], [93].

Another challenge with modelling interactions between fungal effectors and plant proteins may arise from their frequent dependence on a small number of amino acids, exemplified by many known NLR effector pairs [94], [95], [96], [97], [98], [99]. Although binding free energy changes resulting from missense mutations in AlphaFold-multimer complex models are not highly reliable [100], they may be close to those of accurate physics-based methods for AlphaFold3 [69], [100]. The impacts of missense mutation on AlphaFold3 models of effector host protein pairs, to our knowledge, have not been explored.

Several AI techniques devoted to modelling PPIs with greater accuracy have also yet to be applied to EHPCs. For instance, PeSTo can predict potential protein binding regions in protein structures [101], which could supplement AlphaFold3 complex models or help guide experimentation for EHPCs that are difficult to model. Furthermore, AlphaFold-multimer has recently been used for identifying PPIs involving intrinsically disordered proteins [102]. Intrinsic disorder may be a biologically important property of EHPCs [103], [104], and applying existing models to detecting host or effector proteins that may interact with disordered regions may be worth exploring.

One of the main limitations of current AI methods is the substantial computational resources required for making predictions from MSAs. So far, MSA-based methods like AlphaFold3 are the most accurate for PPI analysis. Despite this, MSA-based methodologies can generate poor models of fast-evolving pathogen effectors and plant receptors, many of which have few to no homologues. Models using pLM embeddings have shown promise for faster PPI predictions from single amino acid sequences. Such models may also improve detection of PPIs for effectors or host proteins with sparse homology. However, their accuracy still currently significantly trails that of AlphaFold3 [105].

An alternative approach for detecting new EHPCs is to use generative models like RFdiffusion to sample structures likely to bind an effector or host protein. These generative structures could then be used to search for partial matches to fungal effector or host proteins with fast structural comparison tools like FoldSeek (Fig. 2 B) [106]. This approach would enable researchers with limited computational resources to efficiently screen whole proteomes and detect EHPCs with sparse homology. The advent of online, webserver-based implementations of AI protein structure prediction tools has also improved accessibility.

Recently, RFdiffusion showed promise for identifying host proteins that interact with 5 *M. oryzae* effectors [107]. Structural matches between *O. sativa* and RFdiffusion-generated effector binding proteins were enriched with immunity-related Gene Ontology terms, indicating their biological relevance. To our knowledge, this is also the first study to predict interacting host proteins agnostic to effector function. Given enough computational power, the true diversity of potential PPIs could be explored with broader proteome screens. However, prioritising and experimentally validating PPIs from such screens may be challenging, in addition to determining whether interactions are biologically relevant to phytopathogenic establishment on host plants.

Assessment of biological relevance of EHPC models may be improved by integrating high throughput data describing expression timing and subcellular localisation of effectors and receptors. As described above [73], transcriptomic data have already been used to narrow down PPIs for experimentation based on expression of plant hydrolase-inhibiting effectors during infection. Advancements in single cell and single nucleus sequencing may offer higher resolution analysis of the temporal and spatial patterning of both effector and receptor expression [108]. These techniques have already begun to be applied to plant pathogen interactions. For example, a single cell atlas of gene expression in *A. thaliana* leaves infected with *Colletotrichum higginsianum* showed that Toll/interleukin-1 receptor NLRs were expressed in the vasculature, whereas coiled-coil NLR genes were not [109]. Although spatial proteomic techniques have shown promise for directly determining subcellular localisation of plant proteins [110], they still suffer from numerous technical challenges. High throughput temporal and spatial resolution of effector and receptor expression may start to intersect with large-scale PPI analysis as techniques become more established for plant pathogens.

## 6 Conclusion

AI protein complex modelling is a rapidly developing field. Early adoption of AI models has shown promise for identifying new effector host protein pairs and binding interfaces. However, key challenges remain, such as computational resource requirements, sensitivity to missense mutations and general structural accuracy, especially for fast-evolving effectors and plant proteins with sparse homology. Emerging AI research aimed at improving complex model accuracy and single sequence prediction shows promise for overcoming these challenges. Combining AI methods with expert biochemical interpretation and experimental validation will likely drive the most significant breakthroughs in PPI research. As with many other scientific endeavours, the investigation of PPIs at the molecular interface between plant and fungal pathogen is likely to be profoundly influenced by advancements in AI over the coming years.

## CRediT authorship contribution statement

**C. Verdonk:** Conceptualization, Writing - original draft, Writing – review & editing. **KK Gagalova:** Conceptualization, Writing - original draft, Writing – review & editing. **S. Raffaele:** Writing – review & editing. **MC Derbyshire:** Conceptualization, Writing - original draft, Writing – review & editing.

## Declaration of Competing Interest

None.
